# Validity Analysis of WalkerView^TM^ Instrumented Treadmill for Measuring Spatiotemporal and Kinematic Gait Parameters

**DOI:** 10.3390/s21144795

**Published:** 2021-07-14

**Authors:** Marco Bravi, Carlo Massaroni, Fabio Santacaterina, Joshua Di Tocco, Emiliano Schena, Silvia Sterzi, Federica Bressi, Sandra Miccinilli

**Affiliations:** 1 Unit of Physical Medicine and Rehabilitation, Università Campus Bio-Medico di Roma, via Alvaro Del Portillo 5, 00128 Rome, Italy; m.bravi@unicampus.it (M.B.); f.santacaterina@unicampus.it (F.S.); s.sterzi@unicampus.it (S.S.); f.bressi@unicampus.it (F.B.); s.miccinilli@unicampus.it (S.M.); 2 Unit of Measurements and Biomedical Instrumentation, Università Campus Bio-Medico di Roma, via Alvaro Del Portillo 21, 00128 Rome, Italy; j.ditocco@unicampus.it (J.D.T.); e.schena@unicampus.it (E.S.)

**Keywords:** gait, sensors, biomechanics, instrumented treadmill, contactless kinematics

## Abstract

The detection of gait abnormalities is essential for professionals involved in the rehabilitation of walking disorders. Instrumented treadmills are spreading as an alternative to overground gait analysis. To date, the use of these instruments for recording kinematic gait parameters is still limited in clinical practice due to the lack of validation studies. This study aims to investigate the performance of a multi-sensor instrumented treadmill (i.e., WalkerView^TM^, WV) for performing gait analysis. Seventeen participants performed a single gait test on the WV at three different speeds (i.e., 3 km/h, 5 km/h, and 6.6 km/h). In each trial, spatiotemporal and kinematic parameters were recorded simultaneously by the WV and by a motion capture system used as the reference. Intraclass correlation coefficient (ICC) of spatiotemporal parameters showed fair to excellent agreement at the three walking speeds for steps time, cadence, and step length (range 0.502–0.996); weaker levels of agreement were found for stance and swing time at all the tested walking speeds. Bland–Altman analysis of spatiotemporal parameters showed a mean of difference (MOD) maximum value of 0.04 s for swing/stance time and WV underestimation of 2.16 cm for step length. As for kinematic variables, ICC showed fair to excellent agreement (ICC > 0.5) for total range of motion (ROM) of hip at 3 km/h (range 0.579–0.735); weaker levels of ICC were found at 5 km/h and 6.6 km/h (range 0.219–0.447). ICC values of total knee ROM showed poor levels of agreement at all the tested walking speeds. Bland–Altman analysis of hip ROM revealed a higher MOD value at higher speeds up to 3.91°; the MOD values of the knee ROM were always higher than 7.67° with a 60° mean value of ROM. We demonstrated that the WV is a valid tool for analyzing the spatiotemporal parameters of walking and assessing the hip’s total ROM. Knee total ROM and all kinematic peak values should be carefully evaluated, having shown lower levels of agreement.

## 1. Introduction

Walking represents the primary locomotion modality of the human being. Different pathologies, both musculoskeletal or neurological, may alter this function, causing a restriction of autonomy and participation in everyday life [1,2]. Being a rather complex function, walking disorders present multiple alterations of gait parameters (i.e., spatiotemporal, kinematic, and kinetic) [3]. Therefore, detecting these abnormalities is pivotal for professionals involved in the rehabilitation of walking disorders to set up an appropriate treatment plan and perform a successful rehabilitation program.

Stride assessments, lower limb range of motion (ROM) through joint angular displacements, as well as inter-limb symmetry, are essential features that should be evaluated to properly assess different movement disorders related to diseases such as orthopedics’ disease, Parkinson’s disease, stroke, cerebral palsy [4,5,6,7,8], and to monitor the progress of recovery during rehabilitation.

The literature emphasizes the need to use valid outcome measures to assess walking and offers therapists the ability to monitor the progress of their work in correcting gait alterations [9]. The National Institute for Health and Clinical Excellence also stresses the need to obtain objective quantitative measurements to guarantee quality clinical practice [10]. However, the most common methods for analyzing gait in clinical practice are based on visual observation and questionnaires [9,11,12], which is already demonstrated to be inefficient and poorly reliable [13,14,15].

Currently, clinicians can refer to different types of instruments to carry out an objective gait analysis and obtain quantitative data. The gold standard for measuring spatiotemporal gait parameters (i.e., step and stride length, step and stride time, cadence, and speed) and kinematic variables (e.g., joint angles and angular velocity) is represented by optoelectronic 3D motion capture systems [16,17]. These systems are expensive and require a dedicated laboratory and highly trained personnel to collect and analyze the data in what is typically a time-consuming process [18,19]. To overcome some of these limitations, in recent years, wearable devices have become an alternative to the expensive and strictly lab-based methods for quantifying gait and human movements as extensively reviewed in [20] and [18]. Among others, inertial measurement units (IMUs) have been used in different scenarios because of their advantages of being inexpensive and less bulky than other technologies [21]. IMUs can be used to monitor gait events and spatiotemporal parameters with acceptable accuracy, even using only one sensor [22]. Potentially, more IMUs can be used to register joint kinematics when positioned on specific body landmarks. However, to date, they cannot be considered as a robust solution for monitoring these variables due to the sensor’s output drift, the reproducibility of the sensor’s positioning, and the system calibration, which affect the overall validity of this technology [23,24]. Additional tools are available, such as mats with pressure sensors which appear to have good reliability and repeatability [25], however, these tools still lack kinematic analysis.

In addition to these systems, instrumented treadmills spread as an alternative to overground gait analysis [26]. These systems offer a significant advantage to overground analysis, as they allow the acquisition of a higher number of steps [27]. These systems may be used with two main purposes: tools for gait training and recording both spatiotemporal parameters and kinematic variables using their integrated sensor(s). Among others, WalkerView^TM^ (WalkerView^TM^–WV–by TecnoBody, Bergamo, Italy) is a promising technology that merges signals gathered from the 3D camera, two IMUs on feet, and the eight load cells to retrieve spatiotemporal parameters and kinematic variables in real time with dedicated software.

Although there are numerous studies aimed at validating the use of a single 3D camera for gait analysis on the treadmill [28,29,30] and overground [31,32], there are only a few validation studies related to the instrumented treadmills [33]. There are no validation studies related to WV, thus limiting its use in clinical practice.

The purpose of this study is to validate both the spatiotemporal parameters and kinematic variables retrieved by the WV against those calculated by a reference 3D motion capture system (i.e., MoCap) during walking activity.

## 2. Materials and Methods

### 2.1. Participants

A total of 17 healthy adult participants (7 males -M- and 10 females -F-, details in Table 1) were enrolled for this study. The participants were recruited through flyers posted throughout the University and assessed for eligibility by clinicians of the Unit of Physical and Rehabilitation Medicine at University Campus Bio-Medico. Inclusion criteria were: (1) age > 18 years; and (2) signing the written informed consent. Exclusion criteria were: (1) inability to perform a walking test; and (2) disorders that could affect the execution of the walking tests (e.g., lower limb joint pain, balance disorder, etc.). The study received the Ethical Committee of University Campus Bio-Medico di Roma approval (64.1(18).19 OSS ComEt-UCBM) and was carried out in accordance with the Declaration of Helsinki.

### 2.2. Treadmill System

The instrumented treadmill WV was used in this study. It is equipped with an instrumented belt with eight load cells (load range: 0–150 kg) and a 3D Camera for motion capture (model Kinect v2, Microsoft; acquisition frequency 25 Hz) for sports medicine, rehabilitation, and gait analysis. The treadmill is controlled (e.g., type of trial and assessment module, speed, elevation) by an expert physiotherapist via a control PC with a 15″ touchscreen. A 48″ wide LCD screen allows the physiotherapist to guide the participant during gait training and provides virtual reality and biofeedback at the same time. The dimensions of the WV are L: 2750 mm D: 900 mm H: 2050 mm, with a mass of 280 kg. The WV speed can be set in the range of 0–20 km/h with an incremental step of 0.2 km/h. The integrated software (i.e., TecnoBody Management System, Bergamo, Italy) allows analysis of spatiotemporal parameters (step time, cadence, left and right stance and swing time, left and right step length) and kinematic variables (total ROM, maximum and minimum angle value in the sagittal plane of hip and knee) in real time.

### 2.3. Reference Motion Capture System

**Figure 1:**
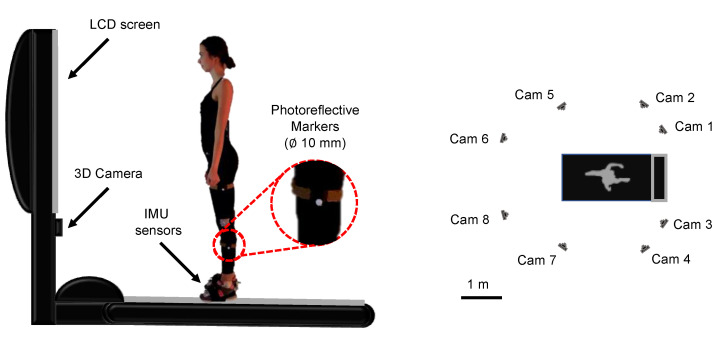
A schematic representation of the instrumented treadmill and of the participant with attached retro-reflective markers and IMU sensors at the foot level (on the left of the figure). A schematic representation of the camera positions around the treadmill (on the right of the figure).

A MoCap was used as the reference system for the overground gait analysis. Eight infrared cameras of the MoCap (SMART D by BTS Bioengineering, Milan, Italy) were positioned around the treadmill (Figure 1). Trajectories of retro-reflective passive markers were recorded at 60 Hz. The inter-laboratory reliability of this system was demonstrated by Benedetti et al. [34].

### 2.4. Experimental Setup and Data Processing

#### 2.4.1. Participant Preparation

**Figure 2:**
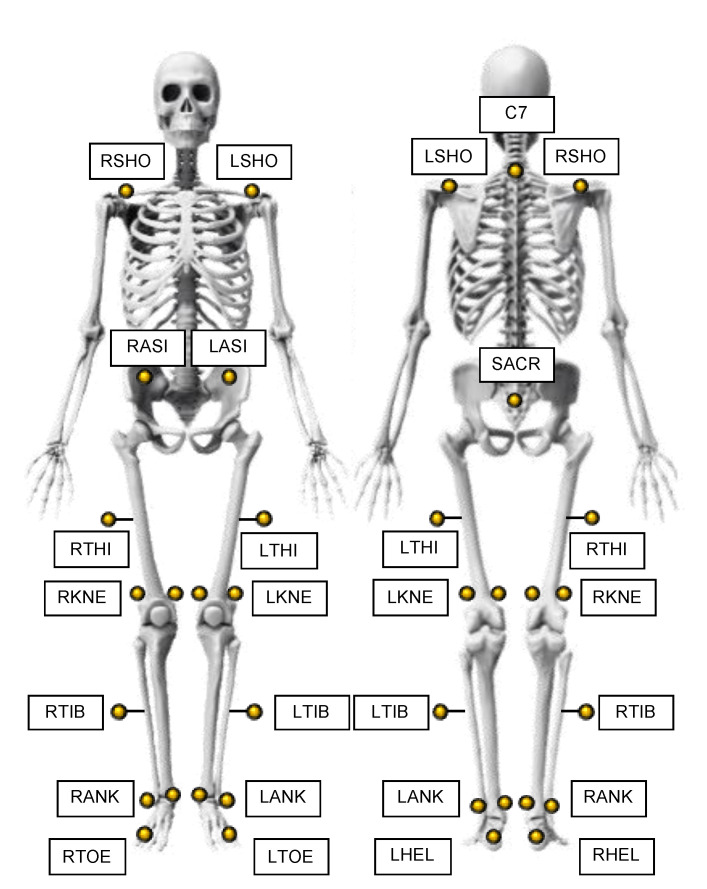
The Conventional Gait Model marker set. **LSHO/RSHO**: Left/Right Shoulder. Markers on Left/Right acromioclavicular joint. **LASI/RASI**: Left/Right ASIS. Markers over Left/Right anterior superior iliac spine. **LTHI/RTHI:** Left/Right thigh. Wand marker over the lateral aspect of the thigh. **LKNE/RKNE:** Left/Right Knee. Markers on lateral epicondyle of the Left/Right knee. **LTIB/RTIB:** Left/Right Tibia. Wand marker over the lateral aspect of the tibia. **LANK/RANK:** Left/Right Ankle. Markers on lateral malleolus along an imaginary line that passes through the trans-malleolar axis. **LTOE/RTOE:** Left/Right Toe. Markers are placed on the lateral aspects of the foot at the fifth metatarsal head. **LHEL/RHEL:** Left/Right Heel. Markers positioned so that the hell-toe marker vector is parallel to the sole of the foot and aligned with the foot progression line. **SACR:** Sacrum. The marker is positioned over the subject’s sacrum. **C7:** 7th Cervical Vertebrae. Markers over the spinous process of the 7th cervical vertebrae.

Retro-reflective markers (diameters of 10 mm) were positioned on all participants by the same physiotherapist with experience in marker setting to avoid potential bias due to inaccurate marker placement [35] (see Figure 2) The Conventional Gait Model marker set (that includes medial markers [36,37]) was used as suggested in the technical manual of the MoCap for gait analysis on the treadmill. This marker set was inspired by the works carried out by Kadaba [36] and Davis [37], respectively, at the Helen Hayes Hospital and Newington Hospital. Furthermore, regarding the definition of the reference system attached to the femur, reference was made to the recommendations of the International Society of Biomechanics [38]. The marker set consists of 22 markers [39], as reported in detail in Figure 1. Height, weight, distance between the anterior iliac spines, pelvis width, lower limb length, knee and ankle diameter of each enrolled participant were collected after the marker placement. These anthropometric measurements were required by the reference MoCap to compute spatiotemporal parameters and kinematic variables. During all the trials, participants wore personal self-selected sneakers. Before gait trials, participants were encouraged to familiarize themselves with the WV and the other equipment through 10 min of walking at self-selected speed [40].

#### 2.4.2. Gait Trials

All participants straddled the WV belt, facing the monitor and were asked to perform a single gait trial for each testing speed (3 km/h, 5 km/h, and 6.6 km/h). The speed of the WV belt followed a trapezoidal pattern: the speed of the belt gradually increased up to the testing speed (in about 10 s), then when the testing speed was reached, 30 s of data were captured for the further spatiotemporal and kinematic gait parameters analysis, and lastly the belt was gradually stopped in about 10 s.

The WV proprietary software automatically calculates spatiotemporal gait parameters and kinematic variables, including steps time (cycles/s), cadence (steps/min), left and right stance time (s), left and right swing time (s), left and right step length (cm), left and right hip ROM (°), left and right hip max angle (°), left and right hip min angle (°), left and right knee ROM (°), left and right knee max angle (°), and left and right knee min angle (°). Per each trial, the treadmill software output is a report with mean and standard deviation values per each variable and parameter, which are then used in the data analysis.

**Figure 3:**
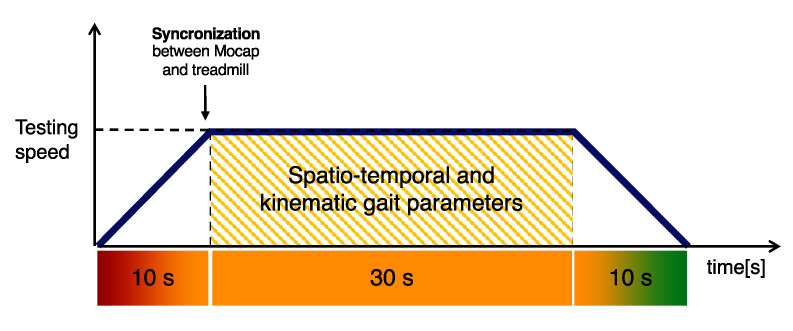
A detailed view of the experimental trial highlighting the 30 s used for the spatiotemporal and kinematic gait parameters data analysis and the synchronization point.

To synchronize the MoCap with the WV data, an additional marker positioned on the left palm was used. This marker was physically covered by closing the hand when the testing speed was reached (see Figure 3). 

In the post-processing phase of the MoCap data, the first heel-strike event after the marker disappearance (see Figure 3 denominated synchronization) was used to identify the following 30 s of data used for the spatiotemporal parameters and kinematic variables analysis (Figure 1, highlighted area). A physical therapist, with experience in motion analysis, manually identified heel-strike and toe-off events through the Smart Analyzer^®^ software (BTS Bioengineering, Milan, Italy). The BTS Bioengineering software was then used to retrieve the same averaged gait parameters from the trajectories of the markers.

### 2.5. Statistical Analysis

The agreement between the two systems was tested by comparing spatiotemporal and kinematic parameters obtained from WV against those recorded by the MoCap. The difference between the WV and MoCap (Difference) and the Root Mean Square Error (RMSE) parameters were calculated per each spatiotemporal and kinematic variable. Intraclass correlation coefficient (ICC) two-way random effects models, absolute agreement, based on single measurements (ICC (2,1)), was computed using SPSS statistical package version 25 (SPSS Inc, Chicago, IL, USA) to evaluate the level of agreement between WV and MoCap. ICC values were interpreted according to the following criteria: values < 0.4 indicate poor agreement; values > 0.4 and <0.6 indicate fair agreement; values > 0.6 and <0.75 indicate good agreement; values ≥ 0.75 indicate excellent agreement [41]. Considering the spatiotemporal and kinematic values from all the systems, the Wilcoxon signed-rank test for paired samples was used to detect significant differences for each variable recorded between the two systems. The Wilcoxon signed-rank test was used since not all the variables were found to be normally distributed through the skewness test. Moreover, the Bland–Altman analysis was carried out per each spatiotemporal and kinematic parameter at each speed, by considering the values recorded by all the volunteers and merging left/right side values. The mean of the difference (MOD) and limit of agreements (LOAs) were calculated and used to investigate the bias between the two instruments [42]. 

## 3. Results

**Figure 4:**
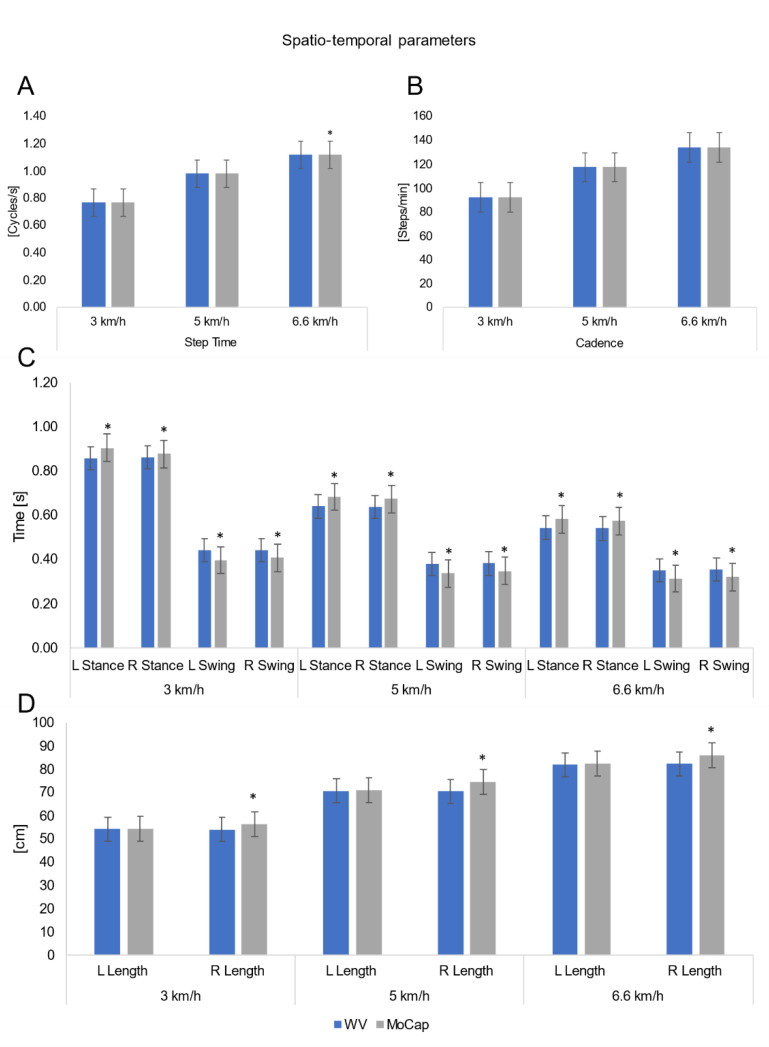
Mean and Standard Deviations (SD) of spatiotemporal parameters. L = left; R= Right. (**A**) Non-significant differences for step time except at 6.6 km/h (*p* < 0.046). (**B**) Cadence recorded by the two systems did not show significant differences. (**C**) WalkerView^TM^ (WV) underestimated stance time and overestimated swing time at all walking speeds (*p* < 0.05). (**D**) WV underestimated R steps length at all walking speeds (*p* < 0.05); non-significant differences for L steps length. Asterisks (*) indicate statistically significant differences (*p* < 0.05-Wilcoxon signed-rank test).

**Figure 5:**
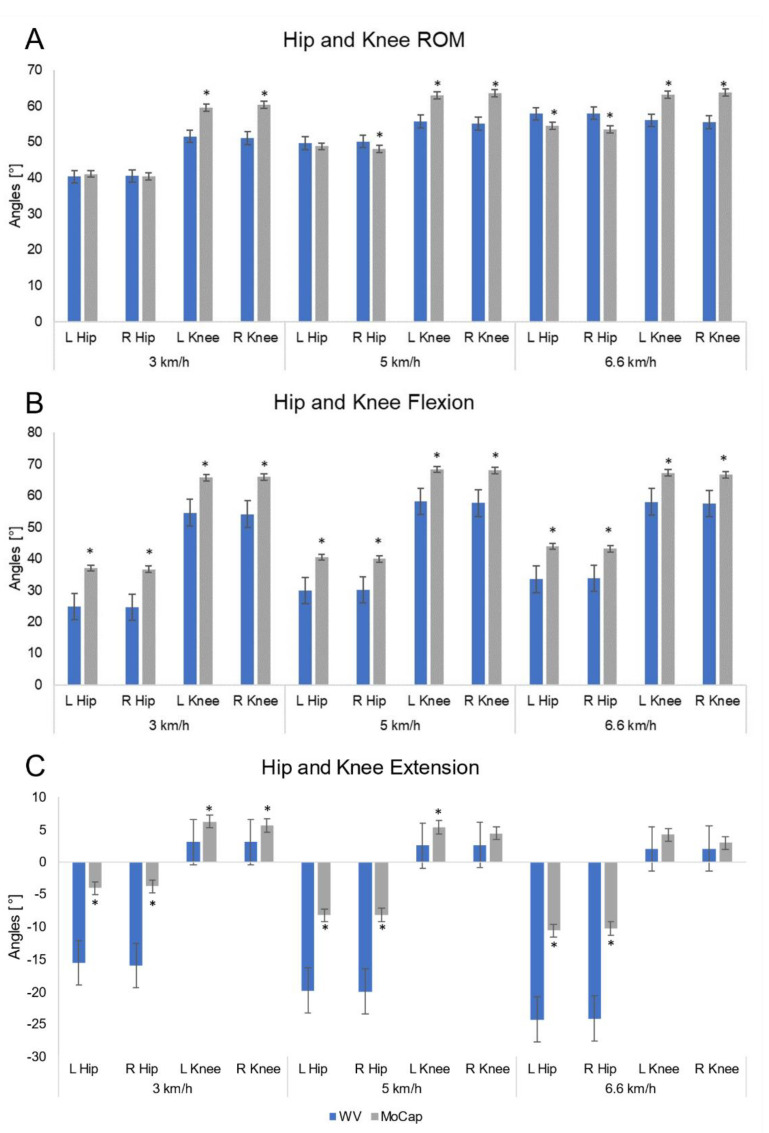
Mean and Standard Deviations (SD) of hip and knee joints total range of motion (ROM). (**A**) Flexion (**B**) and extension (**C**) angular displacements in the sagittal plane at three walking speeds. (**A**) WalkerView^TM^ (WV) overestimated hip ROM at 5 km/h and 6.6 km/h and underestimated knee ROM at all walking speeds. (**B**) WV hip and knee peak flexion were lower than those recorded by MoCap at all walking speeds. (**C**) WV recorded greater hip extension peak measured than MoCap; knee extension peak was underestimated by WV at 3 km/h and only for the left knee at 5 km/h. Asterisks (*) indicate statistically significant differences (*p* < 0.05-Wilcoxon signed-rank test).

**Figure 6:**
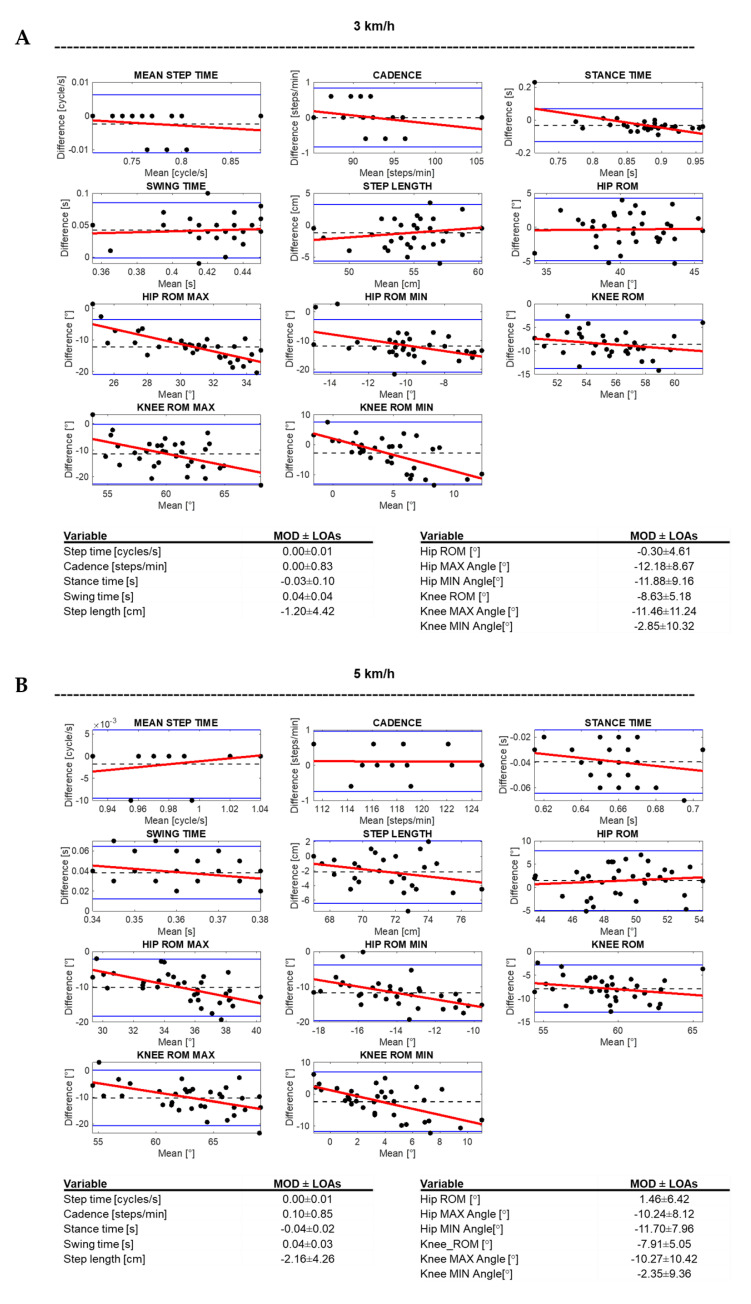

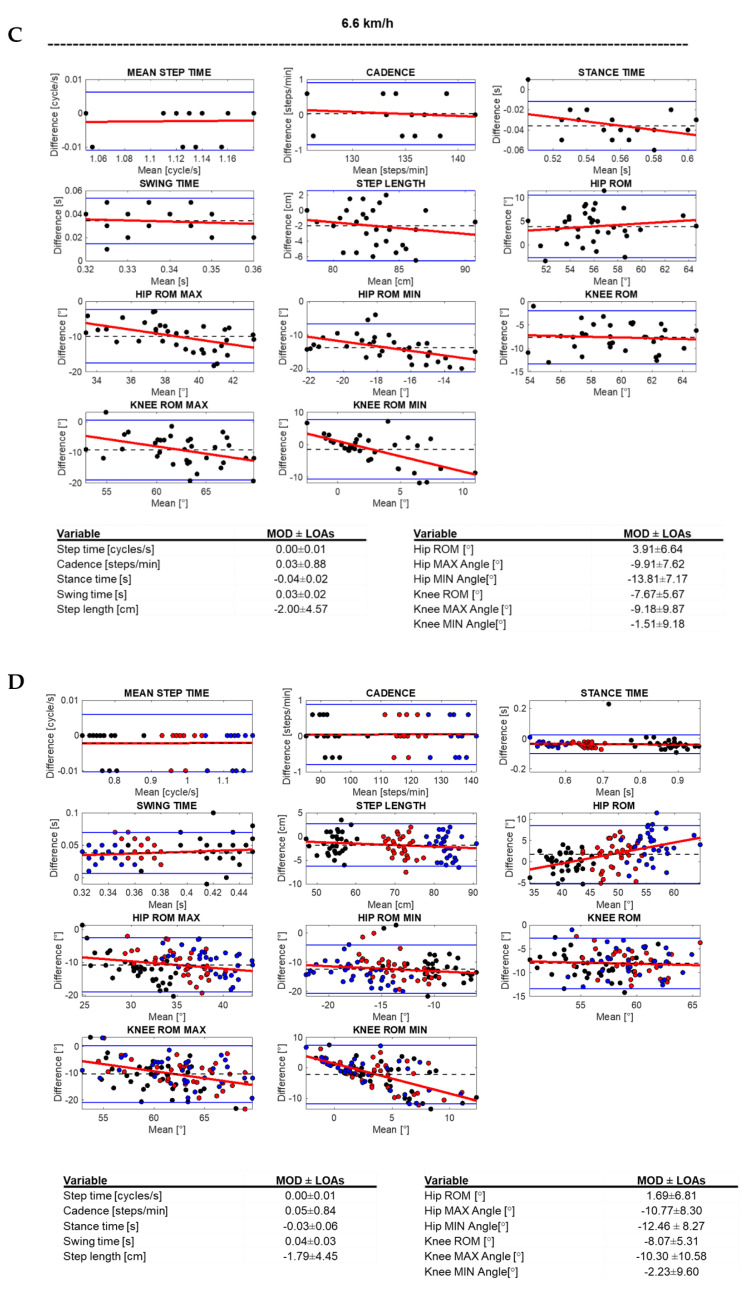
Bland–Altman plots related to the spatiotemporal and kinematical variables recorded from all the volunteers at 3 km/h (**A**), 5 km/h (**B**), and 6.6 km/h (**C**). In the plots, the black dashed line represents the mean of difference (MOD), blue lines represent the limits of agreement (LOA), and red lines show regression line of the difference on means. (**D**) Aggregate values of the three walking speeds: black dots for 3 km/h, red dots for 5 km/h, and blue dots for 6.6 km/h. Below, the tables summarizing the MOD and LOA values.

A total of 51 gait trials were acquired. A minimum number of 21 gait cycles per each volunteer were recorded (mean: 29, max: 36) and post-processed to obtain each spatiotemporal and kinematic value. ICC, *p*-values and the difference between the WV and MoCap are reported in Table 2 and Table 3 and in Figure 4 and Figure 5. Bland–Altman plots, MOD, and LOAs values are shown in Figure 6. 

### 3.1. Spatiotemporal Parameters

Regarding spatiotemporal parameters (Table 2), ICC values showed excellent agreement for mean step time, cadence and left step length at the three tested walking speeds (range 0.768–0.996); fair agreement for right step length, left and right stance time at 3 km/h walking speed (range 0.502–0.596). Weaker levels of agreement were found for the other spatiotemporal parameters (Table 2). The two systems recorded overlapping mean step time and cadence values at all three walking speeds (Figure 4). ICC values showed fair agreement for both left (ICC = 0.594) and right (ICC = 0.502) stance time at 3 km/h, while at higher speed poor agreement (ICC < 0.4) was detected. Poor agreement was also detected for left and right swing time at all walking speeds (ICC < 0.4). 

WV and MoCap left step length demonstrated excellent levels of agreement (ICC > 0.768) at all walking speeds, while fair to poor agreement was found for right step length (ICC range 0.596–0.346) at all walking speeds. As for the left step length, the WV recorded shorter steps and the highest difference (−0.44 ± 1.32 cm; RMSE = 1.36 cm) was recorded at 6.6 km/h. Higher differences were recorded for the right step length and also in this case the WV recorded shorter steps; the highest value (−3.91 ± 1.34 cm; RMSE = 4.12 cm) was recorded at 5 km/h.

For all three walking speeds, MOD values of spatiotemporal parameters were comparable, with a maximum value of 0.04 s for swing/stance time and WV underestimation of 2.16 cm for step length (Figure 6). The analysis of the regression-based Bland–Altman plots reveal that for the spatiotemporal parameters there is no evident proportional bias between the two systems; in fact, in agreement with Ludbrook [43], when the slope of the regression line of the differences on the mean is very close to zero there is no proportional bias.

### 3.2. Kinematic Variables

The WV recorded total ROM and peak of flexion and extension angular displacements of the knee and hip joints. 

Hip total ROM recorded through WV (Table 3) showed non-significant differences, lower than 1°, at 3 km/h, and fair to excellent agreement were found (ICC > 0.5). At higher speeds, higher differences between the two systems were obtained. The ICC levels ranged from the highest (0.604) at 6.6 km/h for the left limb, to the lowest (0.219) recorded for the right limb at 6.6 km/h.

WV knee total ROMs were smaller than those recorded by the MoCap at all the walking speeds. ICC values ranged between 0.098 for right knee at 3 km/h to 0.199 for the left knee at 6.6 km/h showing poor levels of agreement.

Peaks of flexion (max angle) and extension (min angle) angular displacements were consistently different between WV and MoCap in every case (Table 3).

The mean differences and RMSE between systems of the hip ROM on the sagittal plane at 3 km/h, 5 km/h, and 6.6 km/h were respectively −0.74° ± 2.19° RMSE = 2.25°, 0.92° ± 2.85° RMSE = 2.92°, and 3.33° ± 2.86° RMSE 4.33° for the left side and 0.14° ± 2.49° RMSE = 2.42°, 2.01° ± 3.66° RMSE = 4.08°, and 4.49° ± 3.85° RMSE = 5.84° for the right side (Figure 5 and Table 3). Bland–Altman analysis revealed a higher MOD value at higher speeds up to 3.91° (Figure 6). Regression-based Bland–Altman plots reveal the presence of a proportional bias for hip peak of flexion and extension since a negative slope of the regression line is evident (Figure 6.).

Regarding the knee range of motion on the sagittal plane, the following results were obtained, respectively, for 3, 5, and 6.6 km/h: −7.92° ± 2.81° RMSE = 8.38°, −7.31° ± 2.81° RMSE = 7.80°, −7.05° ± 3.02° RMSE = 7.63° for the left side, and −9.33° ± 2.34° RMSE = 9.60°, −8.52° ± 2.23° RMSE = 8.79°, and −8.29° ± 2.71° RMSE = 8.70° for the right side (Table 3 and Figure 5). The MOD values retrieved from the Bland–Altman analysis were always higher than 7.67° (mean value of the ROM was 60°, see Figure 6). The regression lines of the differences on the mean of knee peak of flexion and extension have a negative slope revealing the presence of a proportional bias (Figure 6).

## 4. Discussion

The goal of this study was to evaluate an instrumented treadmill (i.e., WV) in terms of both spatiotemporal and kinematic gait parameters measured on healthy volunteers while walking. The majority of instrumented treadmills do not embed cameras; thus, the kinematical analysis is impossible [33,44]. In fact, kinematic variables are rarely evaluated with instrumented treadmills typically embedding pressure sensors [33] or load cells [45] only. Differently from other instrumented treadmills, the WV merges signals gathered from the 3D camera, the IMUs on feet, and the eight load cells to obtain spatiotemporal parameters and kinematic variables in real time. The WV proprietary software allowed to estimate spatiotemporal parameters and kinematic variables in real time, for the left and right sides separately.

The results demonstrated a Pearson’s coefficient ranging from moderate to very strong levels when spatiotemporal parameters retrieved by the WV were compared against the reference values, as well as MOD values of gait phases up to 0.04 s. In consideration of the ICC levels of agreement and of the low values of RMSE, we can define the WV as a valid tool for analyzing some of the main spatiotemporal parameters (i.e., mean steps time, cadence, and step length and stance time), while for the swing time the same considerations cannot be made having shown a lower level of agreement with the MoCap system. These results are comparable with those presented by Eltoukhy et al. [28] where a single Microsoft Kinect v2 was used for estimating spatiotemporal parameters of healthy volunteers walking at 4.7 and 5.8 km/h on a treadmill. It is necessary to clarify that, differently from what was found in the literature, our analysis was carried out separately for the right and left side of the body and at different walking speeds. No similar studies have been found, so a complete comparison cannot be performed. Regarding the step length, Eltoukhy et al. obtained good to excellent levels of agreement (ICC of 0.76 at 4.7 km/h of walking speed and 0.68 at 5.8 km/h of walking speed) while we obtained ICC values of 0.848 and 0.346 (poor to excellent agreement), respectively, for the left and right side at 5 km/h and 0.854 and 0.381, respectively, for the left and right side at 6.6 km/h. As for step time, Eltoukhy et al. [28] reported an ICC value of 0.87 and 0.82, respectively, at 4.7 and 5.8 km/h while we obtained an ICC value of 0.990 and 0.988, respectively, at 5 and 6.6 km/h of walking speed. These results are also consistent with those reported in a focused review on the validity of the Microsoft Kinect for gait assessment [46]. Our research uses the WV to record spatiotemporal parameters integrating the information obtained from the 3D camera and the load cells, and since the results agreed with what is described in the literature [28,46], the load cells integrated in the treadmill would seem to not add value in terms of validity in the measurement of the spatiotemporal parameters.

Different considerations must be made regarding the kinematic variables. If we consider the ability of WV to assess the total ROM in the sagittal plane of the hip and knee joints, the ICC values obtained are lower than the spatiotemporal parameters. These values seem to be also linked to the walking speed; indeed, at 3 km/h ICC values ranged from fair to excellent agreement for hip total ROM, while weaker values were obtained at 5 km/h and 6.6 km/h of walking speed, while poor agreement was found for knee total ROM. Kinematic results are partially comparable with those presented in the literature. Our total hip ROM results are similar to those of Eltoukhy et al. [26] which reported an excellent level of agreement, while for total knee ROM the results are conflicting. In fact, our poor level of agreement does not reach the good to excellent levels reported by Elthoukhy et al. It should be emphasized that it was only possible to partially compare the data relating to total ROM of the hip and knee since Eltoukhy et al. [26] reported aggregated data between the right and left side while our study reports separate data. Furthermore, conflicting results were found regarding the correlation between ICC values and walking speed, in fact our results suggest that at higher speeds the level of agreement decreases, in contrast to the results of Elthoukhy et al. which, even if moderate, detected an increase in ICC values at a higher walking speed.

The results obtained of knee flexion and extension peak on the sagittal plane are in line with those of Mentiplay et al. [32]; however, we need to clarify that we can partially compare our results with those of Mentiplay et al. In fact, they analyzed only the peaks of the knee and during overground walking, and used Pearson’s correlation coefficient instead of the ICC to evaluate the agreement between the Microsoft Kinect and the MoCap.

The MOD values confirm a substantial agreement between WV and the Mocap system regarding hip total ROM (highest MOD of 3.91° at 6.6 km/h, see Figure 2), while the data relating to knee total ROM, according to ICC values, should be considered carefully as the MOD values are over the limit of a clinically significant difference (highest MOD of −8.63° at 3 km/h, see Figure 2) [35]. At 3 km/h walking speed the ICC values are low and in line with those of Mentiplay et al. [32], while, although remaining low they would seem to increase with increasing speed and the same would seem to be valid for peak values of flexion and extension of the knee. Furthermore, our results show that the 3D camera tends to underestimate the knee flexion and extension values.

As for the hip, the peaks of hip flexion and the peak of hip extension reported low values of ICC not comparable with literature; MOD values confirm the disagreement between the two systems and the values of both the flexion and extension peak are greater than 10° making these WV measurements unreliable. Our results confirm the tendency to underestimate the hip flexion peak and to overestimate the hip extension peak, which, as hypothesized by Xu et al. [29], who examined the differences between a 3D camera and an optoelectronic system, could depend on the knee joint that is captured by the 3D camera sensor in a backward position compared with the reference.

The analysis of the mean angular differences recorded by the two systems regarding the total ROM of the hip is essentially negligible, with an average error of less than 1° at 3 km/h and a maximum error of 5.84° at 6.6 km/h for the right hip. As for the total ROM of the knee, the values exceed the 5° considered a clinically significant difference for a joint angle assessment [35]. This, therefore, must be taken into consideration in the clinical setting when analysing, through the WV, angular variations on the sagittal plane of the knee during walking. The mean difference regarding the maximum hip flexion decreases with the increasing walking speed and varies from a maximum of −12.26° at 3 km/h to a minimum of −9.35° at 6.6 km/h, in line with those of Xu et al. [29]. The same cannot be said for the hip extension peak as our results show values similar to the flexion peak with a maximum average difference between the two systems less than 14°, higher than those of Xu et al. [29], which recorded a maximum average difference in the extension peak less than 7°. On the other hand, by comparing the maximum average differences in the peak of knee flexion, our results show a value less than −11.86°, which is much lower than that reported by Xu et al. of −38.26°. It is necessary to clarify that plantar ankle extension and dorsal flexion data were not compared against the reference values since the WV uses two single inertial sensors that do not allow the calculation of the kinematics of the ankle joint.

There are some limitations which need to be considered when analyzing the results of this study. First of all, we must consider that in our study, with the aim of giving indications about the clinical use of this instrumented treadmill in terms of measurement validity, we conducted a validation analysis by comparing the results generated by the two systems without comparing the raw data. A second point to consider is that we are not aware of the type of algorithms used by the WV and it could be that WV uses insufficient smoothing algorithms, which may have contributed to not properly calculating the flexion and extension peaks, as already reported by Pfister et al. [30]. Further analysis of the raw data and an analysis and a possible improvement of the algorithm used by the WV, could improve the results described in this work. Furthermore, this validation study takes into consideration data obtained on a sample of healthy subjects and it will certainly be necessary to verify whether the same results can be confirmed in pathological walking patterns and on a larger sample. Finally, our study exclusively evaluated the kinematic data on the sagittal plane and the spatiotemporal parameters which are the only ones that the WV records. According to Elthouky et al. [28], the exclusion of the kinematics in the frontal and transverse plane can limit the clinical applicability of the tool.

## 5. Conclusions

The findings of the present study demonstrated that the WV could be used in clinical practice to achieve a rapid and accurate assessment of the mean step time, cadence, and step length, while for swing and stance phases the accuracy is low. Regarding the kinematic parameters, our results do not allow us to identify the WV as a valid alternative to gold standard gait analysis systems. In fact, for the kinematic evaluation of the knee and hip joints in the sagittal plane, as also noted by Xu et al. [29], the Kinect sensor, embedded in WV, seems to be able to record the trend of the joint ROM, but the substantial error of the measurement, especially for peaks of ROM, does not allow us to consider its use for clinical assessment. Nevertheless, WV in consideration of the rapidity and ease of gait analysis execution could be used in daily clinical practice to monitor the trend of the kinematic parameters during a gait training period, indeed, even the Bland–Altman analysis does not substantially show a proportional error such as to prevent its use for monitoring the trend of total hip and knee ROM at the three walking speeds (Figure 6A–C). It is therefore possible to also use WV to analyze asymmetries or alterations of the total ROM whose analysis has shown a good level of agreement.

Clinicians and physiotherapists can benefit from objective data, easily retrieved from WV, relating to gait spatiotemporal parameters and total ROM. This allows them also to identify trends in abnormal kinematic parameters that require further investigation with gold standard systems.

## Figures and Tables

**Table 1:** Characteristics of study participants.

Sample Size (n)	17
Gender	7 M; 10 F
Age [years]	26 ± 4 *
Body mass [kg]	62 ± 9 *
Height [cm]	169 ± 7 *
BMI [kg/m^2^]	21.8 ± 2.1 *

* mean ± standard deviation.

**Table 2:** **Spatiotemporal parameters.** Spatiotemporal parameters obtained from the WV and MoCap systems, at all the tested walking speeds. All the values are reported as mean ± standard deviation, considering the values recorded from all the volunteers. Per each walking speed, the difference between the WV and MoCap (Difference), the Root Mean Square Error (RMSE), the ICC, with 95% CI and relative *p* value, are also reported.

Spatiotemporal Parameters	Walking Speed	WV	MoCap	Difference	RMSE ^1^	ICC (95%CI)	ICC *p*-Value
Mean Steps Time (cycles/s)	3 km/h	0.77 ± 0.04	0.77 ± 0.04	0.00 ± 0.00	0.00	0.996 (0.988–0.998)	<0.001
	5 km/h	0.98 ± 0.03	0.98 ± 0.03	0.00 ± 0.00	0.00	0.990 (0.972–0.996)	<0.001
	6.6 km/h	1.12 ± 0.03	1.12 ± 0.03	0.00 ± 0.00	0.00	0.988 (0.965–0.996)	<0.001
Cadence (steps/min)	3 km/h	92.33 ± 4.40	92.33 ± 4.51	0.00 ± 0.42	0.41	0.996 (0.988–0.998)	<0.001
	5 km/h	117.67 ± 3.33	117.56 ± 3.33	0.11 ± 0.44	0.44	0.991 (0.976–0.997)	<0.001
	6.6 km/h	134 ± 3.92	134.08 ± 3.97	0.04 ± 0.45	0.44	0.994 (0.983–0.998)	<0.001
Left Stance Time (s)	3 km/h	0.86 ± 0.004	0.90 ± 0.05	−0.05 ± 0.02 *	0.05	0.594 (−0.080–0.885)	<0.001
	5 km/h	0.64 ± 0.02	0.68 ± 0.02	−0.04 ± 0.01 *	0.04	0.289 (−0.033–0.703)	<0.001
	6.6 km/h	0.54 ± 0.02	0.58 ± 0.02	−0.04 ± 0.01 *	0.04	0.369 (−0.028–0.774)	<0.001
Right Stance Time (s)	3 km/h	0.86 ± 0.004	0.88 ± 0.09	−0.02 ± 0.07 *	0.07	0.502 (0.057–0.784)	0.017
	5 km/h	0.64 ± 0.02	0.67 ± 0.02	−0.04 ± 0.01 *	0.04	0.360 (−0.057–0.759)	<0.001
	6.6 km/h	0.54 ± 0.02	0.57 ± 0.03	−0.03 ± 0.01 *	0.04	0.393 (−0.74–0.778)	<0.001
Left Swing Time (s)	3 km/h	0.44 ± 0.02	0.40 ± 0.03	0.05 ± 0.02 *	0.02	0.222 (−0.078–0.607)	0.003
	5 km/h	0.38 ± 0.01	0.34 ± 0.02	0.04 ± 0.01 *	0.04	0.123 (−0.040–0.450)	0.003
	6.6 km/h	0.35 ± 0.01	0.31 ± 0.01	0.04 ± 0.01 *	0.04	0.092 (−0.41–0.371)	0.013
Right Swing Time (s)	3 km/h	0.44 ± 0.03	0.41 ± 0.02	0.03 ± 0.02 *	0.04	0.300 (−0.108–0.681)	0.004
	5 km/h	0.38 ± 0.01	0.35 ± 0.01	0.03 ± 0.01 *	0.04	0.108 (−0.053–0.410)	0.019
	6.6 km/h	0.35 ± 0.01	0.32 ± 0.01	0.03 ± 0.01 *	0.04	0.145 (−0.30–0.499)	<0.001
Left Step Length (cm)	3 km/h	54.24 ± 3.29	54.26 ± 2.49	−0.03 ± 2.03	1.97	0.768 (0.462–0.910)	<0.001
	5 km/h	70.53 ± 2.45	70.94 ± 1.98	−0.41 ± 1.20	1.24	0.848 (0.634–0.942)	<0.001
	6.6 km/h	81.76 ± 2.59	82.21 ± 2.40	−0.44 ± 1.32	1.36	0.854 (0.648–0.944)	<0.001
Right Step Length (cm)	3 km/h	54.00 ± 3.06	56.38 ± 2.66	−2.38 ± 1.84 *	2.98	0.596 (−0.080–0.868)	<0.001
	5 km/h	70.41 ± 2.15	74.32 ± 2.47	−3.91 ± 1.34 *	4.12	0.346 (−0.050–0.749)	<0.001
	6.6 km/h	82.24 ± 2.84	85.79 ± 2.56	−3.56 ± 2.08 *	4.09	0.381 (−0.104–0.756)	0.001

^1^ RMSE = Root Mean Square Error; * Wilcoxon signed-rank test *p* < 0.05.

**Table 3:** **Kinematic variables.** Kinematic variables obtained from the WV and MoCap systems, at all the tested walking speeds. All the values are reported as mean ± standard deviation, considering the values from all the volunteers. Per each walking speed, the difference between the WV and MoCap (Difference), the Root Mean Square Error (RMSE), the ICC, with 95% CI and relative *p* value, are also reported.

Kinematic Variables	Walking Speed	WV	MoCap	Difference	RMSE ^1^	ICC (95%CI)	ICC *p*-Value
Left Hip ROM (°)	3 km/h	40.37 ± 3.42	41.11 ± 2.68	−0.74 ± 2.19	2.25	0.735 (0.420–0.894)	<0.001
	5 km/h	49.70 ± 3.57	48.78 ± 2.89	0.92 ± 2.85	2.92	0.604 (0.209–0.834)	0.003
	6.6 km/h	57.86 ± 3.64	54.53 ± 3.18	3.33 ± 2.86 *	4.33	0.447 (−0.095–0.779)	0.002
Left Hip Max angle (°)	3 km/h	24.79 ± 2.24	37.05 ± 4.85	−12.26 ± 4.75 *	13.10	0.034 (−0.042–0.199)	0.201
	5 km/h	29.83 ± 2.51	40.51 ± 4.60	−10.68 ± 4.56 *	11.56	0.048 (−0.052–0.249)	0.165
	6.6 km/h	33.51 ± 2.71	43.98 ± 4.23	−10.47 ± 4.19 *	11.23	0.057 (−0.050–0.273)	0.111
Left Hip Min angle (°)	3 km/h	−15.59 ± 2.56	−4.06 ± 3.96	−11.53 ± 4.66 *	12.38	0.003 (−0.045–0.117)	0.462
	5 km/h	−19.86 ± 2.36	−8.26 ± 3.72	−11.60 ± 4.09 *	12.26	0.017 (−0.034–0.136)	0.294
	6.6 km/h	−24.34 ± 2.51	−10.60 ± 3.68	−13.74 ± 3.52 *	14.16	0.036 (−0.025–0.188)	0.062
Right Hip ROM (°)	3 km/h	40.56 ± 2.29	40.42 ± 3.00	0.14 ± 2.49	2.42	0.579 (0.138–0.825)	0.007
	5 km/h	50.13 ± 3.03	48.12 ± 3.07	2.01 ± 3.66 *	4.08	0.240 (−0.163–0.612)	0.128
	6.6 km/h	58.00 ± 3.71	53.51 ± 3.26	4.49 ± 3.85	5.84	0.219 (−0.117–0.579)	0.053
Right Hip Max angle (°)	3 km/h	24.55 ± 1.40	36.66 ± 4.90	−12.11 ± 4.22 *	12.78	0.048 (−0.040–0.239)	0.103
	5 km/h	30.13 ± 2.15	39.93 ± 4.38	−9.80 ± 3.77 *	10.46	0.080 (−0.052–0.341)	0.049
	6.6 km/h	33.85 ± 2.18	43.21 ± 3.91	−9.35 ± 3.60 *	9.98	0.066 (−0.049–0.301)	0.075
Right Hip Min angle (°)	3 km/h	−16.00 ± 2.21	−3.76 ± 4.10	−12.24 ± 4.80 *	13.09	−0.008 (−0.052–0.097)	0.599
	5 km/h	−20.01 ± 1.98	−8.19 ± 4.06	−11.81 ± 4.16 *	12.48	0.020 (−0.034–0.144)	0.270
	6.6 km/h	−24.19 ± 2.26	−10.30 ± 4.25	−13.89 ± 3.90 *	14.39	0.037 (−0.029–0.195)	0.081
Left Knee ROM (°)	3 km/h	51.58 ± 3.12	59.5 ± 3.31	−7.92 ± 2.81 *	8.38	0.154 (−0.052–0.510)	0.003
	5 km/h	55.73 ± 2.62	63.04 ± 3.37	−7.31 ± 2.81 *	7.80	0.145 (−0.058–0.491)	0.007
	6.6 km/h	56.09 ± 3.01	63.14 ± 3.80	−7.05 ± 3.02 *	7.63	0.199 (−0.070–0.578)	0.003
Left Knee Max angle (°)	3 km/h	54.64 ± 3.49	65.71 ± 6.22	−11.07 ± 6.55 *	12.77	0.046 (−0.079–0.279)	0.268
	5 km/h	58.22 ± 3.57	68.39 ± 6.80	−10.16 ± 6.17 *	11.79	0.131 (−0.092–0.455)	0.074
	6.6 km/h	58.06 ± 4.18	67.30 ± 6.62	−9.24 ± 5.73 *	10.78	0.197 (−0.102–0.560)	0.026
Left Knee Min angle (°)	3 km/h	3.04 ± 2.26	6.21 ± 5.56	−3.18 ± 5.72 *	6.39	0.075 (−0.285–0.479)	0.358
	5 km/h	2.49 ± 2.09	5.35 ± 5.09	−2.86 ± 4.93 *	5.58	0.161 (−0.214–0.547)	0.217
	6.6 km/h	1.98 ± 2.09	4.16 ± 5.24	−2.18 ± 5.15	5.45	0.151 (−0.273–0.558)	0.254
Right Knee ROM (°)	3 km/h	51.08 ± 2.25	60.41 ± 3.23	−9.33 ± 2.34 *	9.60	0.098 (−0.028–0.392)	0.002
	5 km/h	55.12 ± 2.67	63.64 ± 3.05	−8.52 ± 2.23 *	8.79	0.129 (−0.031–0.465)	0.001
	6.6 km/h	55.51 ± 3.19	63.81 ± 2.70	−8.29 ± 2.71 *	8.70	0.118 (−0.044–0.438)	0.006
Right Knee Max angle (°)	3 km/h	54.16 ± 2.70	66.02 ± 4.43	−11.86 ± 4.95 *	12.80	0.014 (−0.045–0.148)	0.364
	5 km/h	57.67 ± 3.58	68.05 ± 5.17	−10.38 ± 4.50 *	11.26	0.132 (−0.067–0.461)	0.020
	6.6 km/h	57.57 ± 3.88	66.70 ± 5.02	−9.13 ± 4.41 *	10.08	0.170 (−0.079–0.528)	0.014
Right Knee Min angle (°)	3 km/h	3.08 ± 2.74	5.61 ± 5.22	−2.54 ± 4.94	5.42	0.262 (−0.154–0.630)	0.114
	5 km/h	2.57 ± 2.84	4.41 ± 4.71	−1.84 ± 4.70	4.92	0.250 (−0.197–0.631)	0.142
	6.6 km/h	2.05 ± 2.84	2.89 ± 4.68	−0.84 ± 4.22	4.18	0.411 (−0.071–0.737)	0.047

^1^ RMSE = Root Mean Square Error; * Wilcoxon signed-rank test *p* < 0.05.

## Data Availability

The data presented in this study are available on request from the corresponding author. The data are not publicly available due to privacy reasons.

## References

[1] Nadeau S., Betschart M., Bethoux F. (2013). Gait analysis for poststroke rehabilitation: The relevance of biomechanical analysis and the impact of gait speed. Phys. Med. Rehabil. Clin. N. Am..

[2] McClelland J.A., Webster K.E., Feller J.A. (2007). Gait analysis of patients following total knee replacement: A systematic review. Knee.

[3] Lacquaniti F., Ivanenko Y.P., Sylos-Labini F., La Scaleia V., La Scaleia B., Willems P.A., Zago M. (2017). Human locomotion in hypogravity: From basic research to clinical applications. Front. Physiol..

[4] DeLuca P.A., Davis R.B., Ounpuu S., Rose S., Sirkin R. (1997). Alterations in Surgical Decision Making in Patients with Cerebral Palsy Based on Three-Dimensional Gait Analysis. J. Pediatr. Orthop..

[5] Kay R.M., Dennis S., Rethlefsen S., Reynolds R.A.K., Skaggs D.L., Tolo V.T. (2000). The effect of preoperative gait analysis on orthopaedic decision making. Clin. Orthop. Relat. Res..

[6] Cook R.E., Schneider I., Hazlewood M.E., Hillman S.J., Robb J.E. (2003). Gait analysis alters decision-making in cerebral palsy. J. Pediatr. Orthop..

[7] Bortone I., Trotta G.F., Brunetti A., Cascarano G.D., Loconsole C., Agnello N., Argentiero A., Nicolardi G., Frisoli A., Bevilacqua V. (2017). A novel approach in combination of 3D gait analysis data for aiding clinical decision-making in patients with Parkinson’s disease. Proceedings of the Lecture Notes in Computer Science (Including Subseries Lecture Notes in Artificial Intelligence and Lecture Notes in Bioinformatics).

[8] Pawik Ł., Fink-Lwow F., Pajchert Kozłowska A., Szelerski Ł., Żarek S., Górski R., Pawik M., Urbanski W., Reichert P., Morasiewicz P. (2021). Assessment of gait after treatment of Tibial nonunion with the Ilizarov method. Int. J. Environ. Res. Public Health.

[9] Coutts F. (1999). Gait analysis in the therapeutic environment. Man. Ther..

[10] Marks D.F. (2002). Perspectives on Evidence-Based Practice.

[11] Lindemann U. (2020). Spatiotemporal gait analysis of older persons in clinical practice and research: Which parameters are relevant?. Z. Gerontol. Geriatr..

[12] Toro B., Nester C.J., Farren P.C. (2003). The Status of Gait Assessment among Physiotherapists in the United Kingdom. Arch. Phys. Med. Rehabil..

[13] Saleh M., Murdoch G. (1985). In defence of gait analysis. Observation and measurement in gait assessment. J. Bone Joint Surg. Br..

[14] Brunnekreef J.J., van Uden C.J.T., Van Moorsel S., Kooloos J.G.M. (2005). Reliability of videotaped observational gait analysis in patients with orthopedic impairments. BMC Musculoskelet. Disord..

[15] Ong A.M.L., Hillman S.J., Robb J.E. (2008). Reliability and validity of the Edinburgh Visual Gait Score for cerebral palsy when used by inexperienced observers. Gait Posture.

[16] Galna B., Barry G., Jackson D., Mhiripiri D., Olivier P., Rochester L. (2014). Accuracy of the Microsoft Kinect sensor for measuring movement in people with Parkinson’s disease. Gait Posture.

[17] Zhang Y., Wang M., Awrejcewicz J., Fekete G., Ren F., Gu Y. (2017). Using Gold-standard Gait Analysis Methods to Assess Experience Effects on Lower-limb Mechanics During Moderate High-heeled Jogging and Running. J. Vis. Exp..

[18] Benson L.C., Clermont C.A., Bošnjak E., Ferber R. (2018). The use of wearable devices for walking and running gait analysis outside of the lab: A systematic review. Gait Posture.

[19] Simon S.R. (2004). Quantification of human motion: Gait analysis—Benefits and limitations to its application to clinical problems. J. Biomech..

[20] Muro-de-la-Herran A., García-Zapirain B., Méndez-Zorrilla A. (2014). Gait analysis methods: An overview of wearable and non-wearable systems, highlighting clinical applications. Sensors.

[21] Massaroni C., Di Tocco J., Raiano L., Carnevale A., Sabbadini R., Lo Presti D., Bravi M., Miccinilli S., Sterzi S., Formica D. Influence of torso movements on a multi-sensor garment for respiratory monitoring during walking and running activities. Proceedings of the 2020 IEEE International Instrumentation and Measurement Technology Conference (I2MTC).

[22] Caldas R., Mundt M., Potthast W., de Lima Neto F.B., Markert B. (2017). A systematic review of gait analysis methods based on inertial sensors and adaptive algorithms. Gait Posture.

[23] Bravi M., Gallotta E., Morrone M., Maselli M., Santacaterina F., Toglia R., Foti C., Sterzi S., Bressi F., Miccinilli S. (2020). Concurrent Validity and Inter Trial Reliability of a Single Inertial Measurement Unit for Spatial-Temporal Gait Parameter Analysis in Patients with Recent Total Hip or Total Knee Arthroplasty. Gait Posture.

[24] De Ridder R., Lebleu J., Willems T., De Blaiser C., Detrembleu C., Roosen P. (2019). Concurrent Validity of a Commercial Wireless Trunk Tri-Axial Accelerometer System for Gait Analysis. J. Sport Rehabil..

[25] Veilleux L.N., Robert M., Ballaz L., Lemay M., Rauch F. (2011). Gait analysis using a force-measuring gangway: Intrasession repeatability in healthy adults. J. Musculoskelet. Neuronal Interact..

[26] Van K.A., Thomson A., Burnett A. (2019). Reliability and validity of the Zebris FDM-THQ instrumented treadmill during running trials. Sport. Biomech..

[27] Sloot L.H., van der Krogt M.M., Harlaar J. (2014). Effects of adding a virtual reality environment to different modes of treadmill walking. Gait Posture.

[28] Eltoukhy M., Oh J., Kuenze C., Signorile J. (2017). Improved kinect-based spatiotemporal and kinematic treadmill gait assessment. Gait Posture.

[29] Xu X., McGorry R.W., Chou L.-S., Lin J.-h., Chang C.-c. (2015). Accuracy of the Microsoft Kinect^TM^ for measuring gait parameters during treadmill walking. Gait Posture.

[30] Pfister A., West A.M., Bronner S., Noah J.A. (2014). Comparative abilities of Microsoft Kinect and Vicon 3D motion capture for gait analysis. J. Med. Eng. Technol..

[31] Clark R.A., Bower K.J., Mentiplay B.F., Paterson K., Pua Y.H. (2013). Concurrent validity of the Microsoft Kinect for assessment of spatiotemporal gait variables. J. Biomech..

[32] Mentiplay B.F., Perraton L.G., Bower K.J., Pua Y.H., McGaw R., Heywood S., Clark R.A. (2015). Gait assessment using the Microsoft Xbox One Kinect: Concurrent validity and inter-day reliability of spatiotemporal and kinematic variables. J. Biomech..

[33] McSweeney S.C., Reed L.F., Wearing S.C. (2020). Reliability and minimum detectable change of measures of gait in children during walking and running on an instrumented treadmill. Gait Posture.

[34] Benedetti M.G., Merlo A., Leardini A. (2013). Inter-laboratory consistency of gait analysis measurements. Gait Posture.

[35] McGinley J.L., Baker R., Wolfe R., Morris M.E. (2009). The reliability of three-dimensional kinematic gait measurements: A systematic review. Gait Posture.

[36] Kadaba M.P., Ramakrishnan H.K., Wootten M.E. (1990). Measurement of Lower Extremity Kinematics During Level Walking. J. Orthop. Res..

[37] Davis R.B., Õunpuu S., Tyburski D., Gage J.R. (1991). A gait analysis data collection and reduction technique. Hum. Mov. Sci..

[38] Wu G., Siegler S., Allard P., Kirtley C., Leardini A., Rosenbaum D., Whittle M., D’Lima D.D., Cristofolini L., Witte H. (2002). ISB recommendation on definitions of joint coordinate system of various joints for the reporting of human joint motion—Part I: Ankle, hip, and spine. J. Biomech..

[39] Stief F. (2016). Variations of marker sets and models for standard gait analysis. Handb. Hum. Motion.

[40] Van De Putte M., Hagemeister N., St-Onge N., Parent G., De Guise J.A. (2006). Habituation to treadmill walking. Biomed. Mater. Eng..

[41] Cicchetti D.V., Sparrow S.A. (1981). Developing criteria for establishing interrater reliability of specific items: Applications to assessment of adaptive behavior. Am. J. Ment. Defic..

[42] Altman D.G., Bland J.M. (1983). Measurement in Medicine: The Analysis of Method Comparison Studies. J. R. Stat. Soc. Ser. D.

[43] Ludbrook J. (1997). Comparing methods of measurement. Clin. Exp. Pharmacol. Physiol..

[44] Reed L.F., Urry S.R., Wearing S.C. (2013). Reliability of spatiotemporal and kinetic gait parameters determined by a new instrumented treadmill system. BMC Musculoskelet. Disord..

[45] Edginton K.A., Güler H.C., Ober J.J., Berme N. (2007). Instrumented treadmills: Reducing the need for gait labs. Comput. Sci..

[46] Springer S., Seligmann G.Y. (2016). Validity of the kinect for gait assessment: A focused review. Sensors.

